# What Happened in That Pit? An Archaeozoological and GIS Approach to Study an Accumulation of Animal Carcasses at the Roman Villa of Vilauba (Catalonia)

**DOI:** 10.3390/ani11082214

**Published:** 2021-07-26

**Authors:** Lídia Colominas, Pere Castanyer, Joan Frigola, Joaquim Tremoleda

**Affiliations:** 1 GIAP Team, Institut Català d’Arqueologia Clàssica, 43003 Tarragona, Spain; 2 Grup de Recerca Arqueològica del Pla de l’Estany, 17820 Banyoles, Spain; pcastanyer@gencat.cat (P.C.); joanfrigola@hotmail.com (J.F.); jtremoleda@gencat.cat (J.T.)

**Keywords:** cattle herd, disease, meat preserves, Roman economy, faunal remains, contextual taphonomy, Iberian Peninsula

## Abstract

**Simple Summary:**

We present a methodological approach to the study of ancient accumulations of animal bones that combines archaeozoological and geographic information system (GIS) analyses. This combined approach was applied to the study of 783 cattle remains recovered in a 187 m^2^ pit at the Roman villa of Vilauba (Catalonia). Its detailed study allowed the nature and formation of this singular assemblage to be documented. We propose that these remains correspond to the carcasses of 14 cattle. They may have contracted some kind of disease, and it was decided to slaughter them in order to take advantage of their meat by preserving it. The study of this exceptional assemblage opens a window onto an unusual and isolated moment of the lives of the inhabitants of this villa, and shows the importance of cattle in its economy.

**Abstract:**

Some of the deposits of animal remains documented throughout prehistory and history are clearly something other than ordinary waste from meat consumption. For the Roman period and based on their characteristics, these assemblages have been classified as butchery deposits, raw material deposits, deposits created for the hygienic management and disposal of animal carcasses, or ritual deposits. However, some are difficult to classify, and the parameters that define each of them are not clear. Here, we present a unique deposit from the Roman villa of Vilauba (Catalonia). A total of 783 cattle remains were found in an irregular-shaped 187 m^2^ pit originally dug to extract the clay used in the construction of the villa walls around the third quarter of the 1st century AD. The application of a contextual taphonomy approach, with the integration of archaeozoological variables, stratigraphy and context, and a GIS analysis, allowed us to document the nature and formation of this singular assemblage. It consisted of the carcasses of 14 cattle individuals from which the meat had been removed to take advantage of it by preserving it. Therefore, the parameters that characterise the refuse of this activity are presented here as a baseline for other studies.

## 1. Introduction

Bones from archaeological sites are the remnants of a wide range of activities. They are usually refuse from domestic meat consumption, although they can also be butchery deposits; waste from activities linked to hide preparation, bone and antler working, or glue manufacture; deposits created for the hygienic management and disposal of animal carcasses; or ritual deposits, to name only some of the possibilities. During the Roman period, large deposits of bones were accumulated as waste from those activities. Several guidelines to define their characteristics have been proposed to help identify the activity that generated them.

Large numbers of terminal limb elements (carpals, tarsals, metapodials, and phalanges), in association with a high proportion of heads, are considered indicative of waste from primary butchery [1,2,3]. In contrast, a preponderance of fractured large bones, scapulae, pelvises, vertebrae, and ribs has generally been considered an indication of domestic waste [2,4]. Once the animal had been slaughtered, however, several products other than meat were of interest for a variety of productions. An abundance of horns, feet, and tails has been associated with tanneries and hide preparation [5,6]. A large number of antlers and fully fused sawn metapodials, accompanied by half-finished artefacts, has been related to antler and bone working [7,8,9,10,11,12]. Deposits composed of diaphyses from systematically broken long bones have been proposed as the refuse from glue manufacture and marrow extraction [6,13,14,15]. Other deposits contain entire animal carcasses, with no evidence of anthropic modification. These have been interpreted as a deliberately symbolic acts, or as a means of disposing of dead animals whose carcasses were not processed, either because the animals were ill when they died or because their meat was not generally eaten (e.g., dogs and equids during the Roman period) [13,16].

This summarised list is obviously very simplistic, and the true picture at any site is likely to have been far more complex and possibly include more than one origin. An example of this complexity is the case presented in this paper. Here, we present a deposit of 783 cattle remains that filled a 187 m^2^, irregularly shaped hole located in front of the Roman villa of Vilauba (Catalonia). The characteristics of this assemblage do not match any previously presented parameters. At the same time, an assemblage consisting mainly of cattle remains such as the one presented here had never been documented at the site. To understand the nature and formation of this singular assemblage, and therefore elucidate the economic importance of this species for the inhabitants of the villa, we undertook a detailed archaeozoological study combined with a GIS analysis. This approach allowed us to answer key questions in the final interpretation of the deposit, such as the degree of homogeneity in the assemblage, the primary or secondary deposition of the remains, and the degree of completeness of the carcasses. This enabled hypotheses to be put forward on the activity that could have generated it, and a baseline to be created for future studies.

## 2. The Site of Vilauba

**Figure 1:**
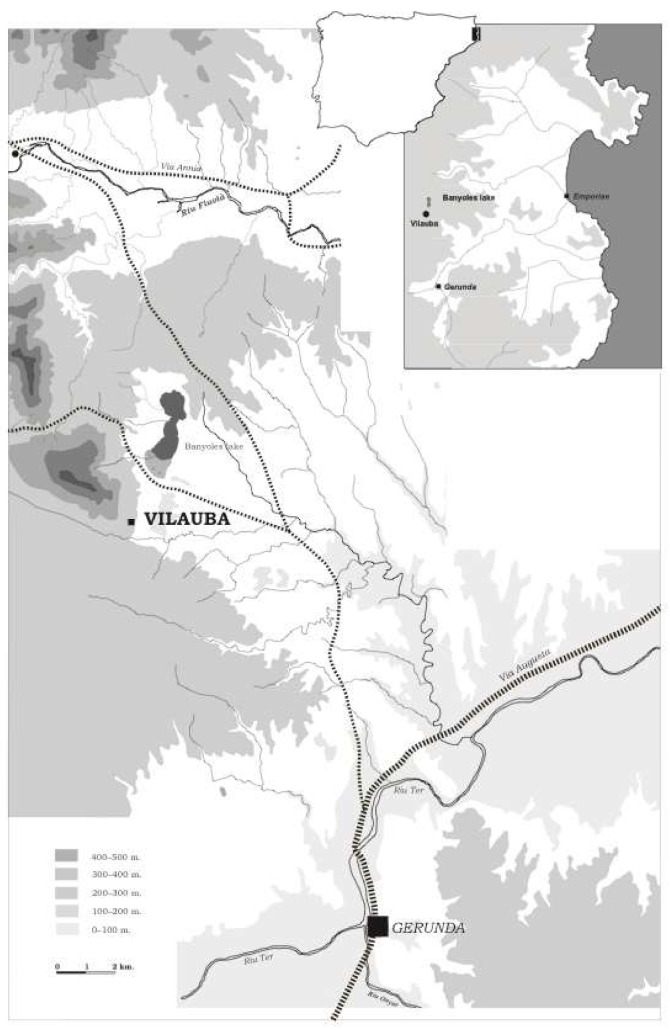
Location of Vilauba in relation to the most important Roman towns in the area.

The villa of Vilauba is in a small valley 3 km south of Lake Banyoles (Girona, Catalonia) (Figure 1). Thorough scientific research has revealed more than 5000 m^2^ of archaeological remains spanning from the 2nd–1st century BC to the 7th century AD [17].

The aim of the Vilauba Roman villa archaeological research project is the comprehensive excavation of this rural establishment. This will help add to our knowledge of the forms of occupation and exploitation of this territory between the early Roman occupation and the end of the ancient world.

**Figure 2:**
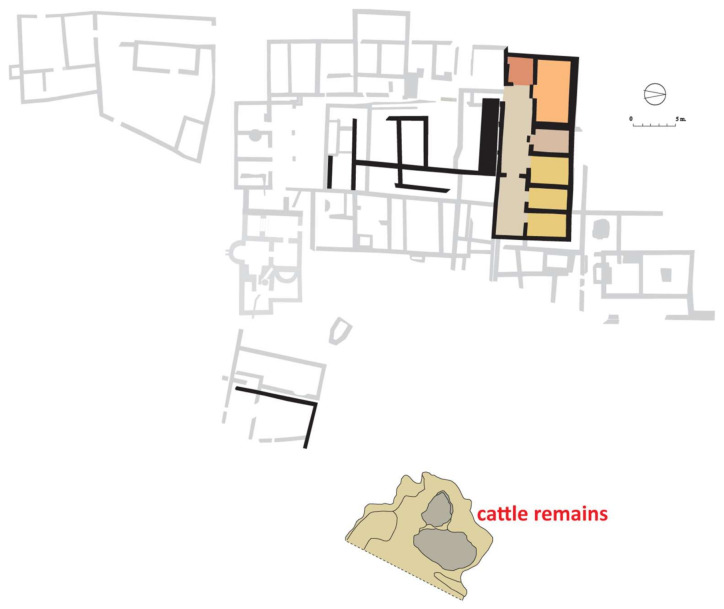
Ground plan of the villa and location of the pit during the third quarter of the 1st century AD.

Despite the attested evidence of occupation prior to the turn of era, the first well-known stage of the archaeological site corresponds to a building from the 1st century AD that was successively reformed and in use until the end of the 3rd century AD. From the first building we know mainly of the residential area with a rectangular ground plan. It was divided longitudinally into two halves, corresponding to a corridor or frontal gallery and a row of six rooms in the rear (pantry/kitchen, triclinium, sacellum, and three bedrooms). To the south, other rooms surrounded a small courtyard or open area whose function is more difficult to attribute [17] (pp. 55–76) (Figure 2). The working area was separated from the residence and has been interpreted as livestock stables and facilities for farming-related activities [17] (pp. 102–105).

The study of these two sectors shows that Vilauba, although it was a modest villa, would have been quite important in its territory, with a theoretical farmed area of between 50 and 85 hectares in this early Roman phase [18] (p. 20). The different seed species identified (wheat, barley, olive, grape, pea, and lentil) suggest that the villa’s lands were farmed throughout the annual cycle. Wheat and barley would have been sown in autumn and legumes planted in spring [19]. At the same time, archaeozoological studies show that sheep, goats, pigs, and cattle were bred at the villa. These animals would have been used mainly to provide wool, milk, meat, and traction [20] (pp. 77–92). They would also have provided fertiliser for the fields. Thus, the inhabitants of the villa of Vilauba would have practised a diversified economy characterised by a combination of crop cultivation and stockbreeding.

### 2.1. Stratigraphy of the Pit and Its Relation to the Villa’s Occupational Sequence

Although the general periodisation of the Vilauba site can be summarised in three major stages, the archaeological record shows that the final ground plan of each stage was the result of a much more complex process that often involved significant reforms and modifications within each period. Thus, the early Roman villa (1st–3rd centuries AD) should be understood as the final result of a process that began much earlier with the construction of a small residential building in the northern sector that occupied a large rectangle some 25 m long and 9 m wide [17] (pp. 55–76), [21] (p. 36). The finds excavated below the current use levels that can be associated with the prior levelling of the terrain allow the construction of this north wing to be dated to around 60 AD. It is precisely considering the dating of this sector of the residential villa that we can associate its construction, both stratigraphically and chronologically, with the large pit where the bone remains we will analyse in detail in this study were found (Figure 2).

**Figure 3:**
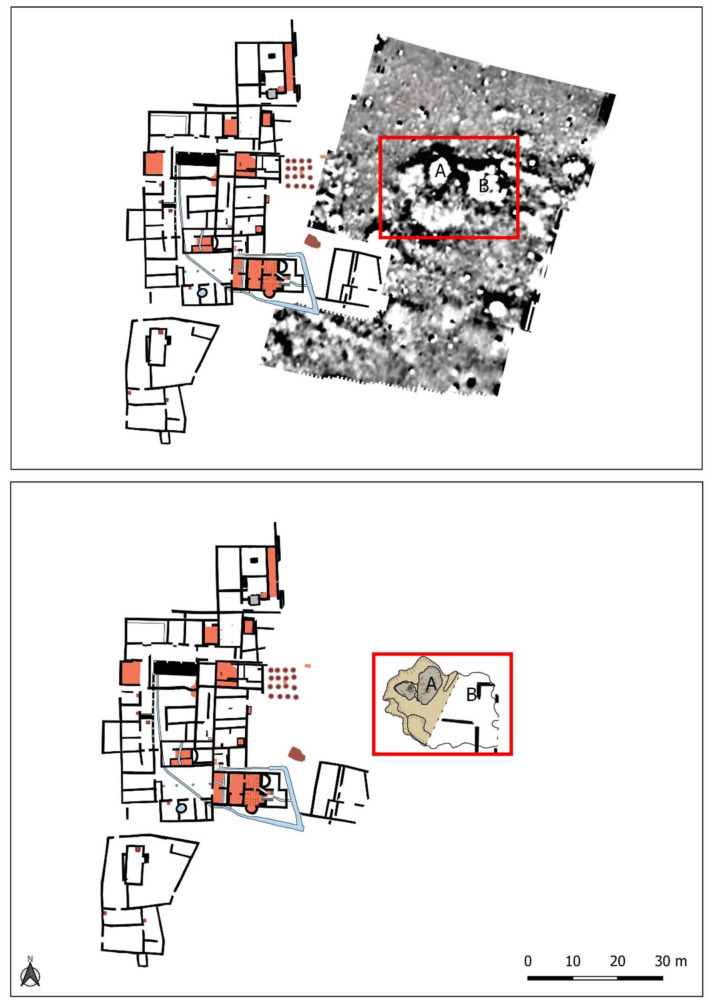
Location of the anomalies A and B detected by geophysical survey. Only anomaly A has been excavated till the date.

The pit was discovered during the geophysical survey carried out in the eastern part of the site (Figure 3). It detected various anomalies, including, of particular note, Groups A and B, which were part of the same complex with a very high magnetic signal bordered by a large negative cut. The remarkable size of this cut, 18 m long and a minimum of 13 m wide, meant that it was first necessary to excavate Anomaly A, which was larger and deeper, and leave Anomaly B for future campaigns [22] (p. 226).

**Figure 4:**
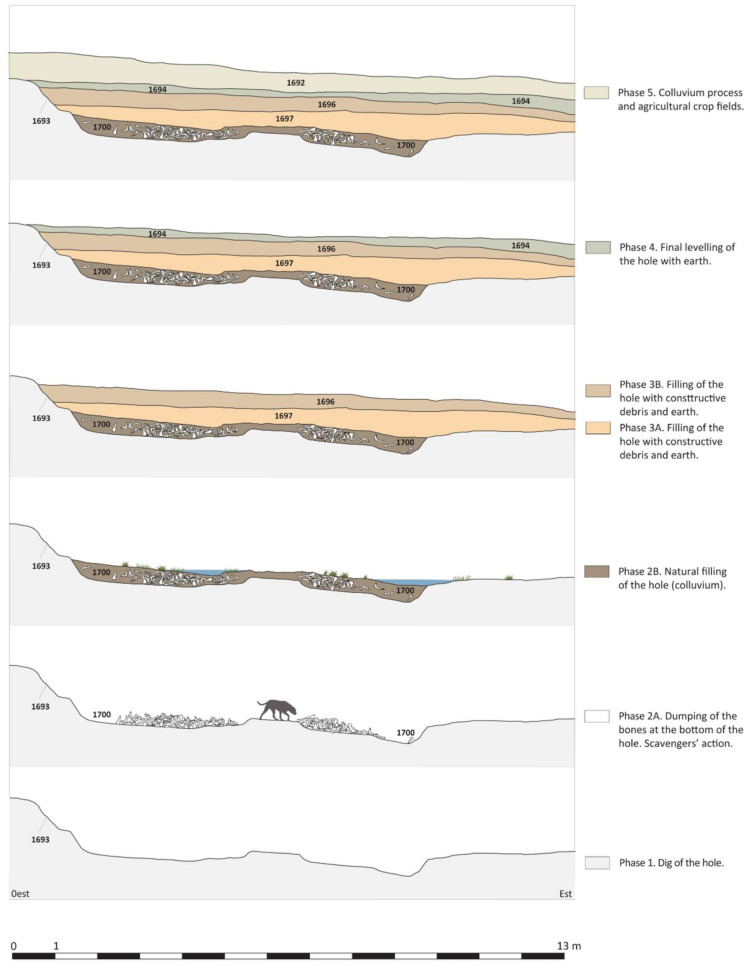
Section of the pit showing how the different stratigraphic units were formed.

The pit was more or less round in shape, although its outline was quite irregular. Stratigraphically, its borders could be perfectly defined, as the soil of the inner backfill contrasted clearly with the colour and composition of the clays that make up the natural sedimentation (Figure 4, U.S. 1693). The excavation made it possible to identify the different stratigraphic units that formed a brief sequence of up to four superimposed levels that could be clearly differentiated from each other and had a total depth of 120 cm. In addition to the surface level removed by more recent agricultural work, these stratigraphic units were identified by record numbers 1694, 1696, 1697, and 1700 (Figure 4).

The study of the characteristics and composition of the stratigraphies and finds allowed us to reconstruct different phases of the process of the pit’s backfill. The three upper stratigraphic units (U.S. 1694, 1696, and 1697) contained large amounts of archaeological finds, especially pottery, as well as pieces of glass, metal, and other materials. In contrast, the lower level (U.S. 1700) was formed mainly of a large number of faunal remains covered by colluvial clays. The study of the characteristics and composition of the stratigraphy and finds allowed us to reconstruct different phases of the process of the pit’s backfill. The three upper stratigraphic units (U.S. 1694, 1696, and 1697) contained large amounts of archaeological finds, especially pottery, as well as pieces of glass, metal, and other materials. In contrast, the lower level (U.S. 1700) was formed mainly of a large number of faunal remains. Most of these remains were from large animals that had apparently been dumped at random, although some fragments still maintained their anatomical connection (Figure 4, Phase 2A). Although the bone remains formed perfectly differentiable groupings, some fragments appeared more dispersed and mixed with the same colluvial clays that also covered the larger concentrations. The presence of these clays indicates stratigraphically that after the bone remains had been dumped, the pit had been left open for a short time, allowing for the deposition of the colluvial level (Figure 4, Phase 2B). Although fewer archaeological finds were made among the bone remains, the absence of pottery of African origin, which was present in the higher levels, allows us to place its formation around the years 50–60 AD.

The dumping of these bone remains (U.S. 1700) thus stratigraphically marks the beginning of the backfill of a large open pit dug in the natural terrain near the villa. The characteristics and size of this enormous hole, as well as the absence of any trace of interior preparation, make any interpretation linked to the Roman villa’s agricultural activities impossible. The hypothesis that seems most feasible to us is that it was dug to obtain the clay needed to build the walls of the residential building and, more specifically, the villa’s north wing (Figure 2 and Figure 4, Phase 1). The natural sedimentation on which the villa of Vilauba was built, formed by clayey silts of a beige-green colour, is especially suitable for building walls, one of the parts of the work that required the greatest volume of material. Use of the natural resources offered by the surrounding area, including for construction, was, according to the ancient Latin agronomists, one of the fundamental precepts of Roman villas. The proximity of this pit to the building would have helped optimise the work and avoid transportation costs.

Above the lower level, the backfill of the pit was formed by two intermediate stratigraphies (1696 and 1697) with a very similar composition and chronology (Figure 4, Phases 3A and 3B). From its excavation, we can highlight the large amount of building waste, iron slag, and pottery, with a wide repertory of common ware, cookware and wine, and olive oil and salted fish amphorae. Some tableware and imports were also found, with a clear predominance of Hispanic and southern Gallic Samian ware. There were also some pieces of fine-walled and North African pottery, particularly cookware, but also some of the first forms of African A tableware (Hayes 3A and 8A). This allows us to place the terminus post quem of this stratigraphic unit around the last decade of the 1st century AD. Finally, the upper level (U.S. 1694) corresponds to a last levelling of the pit, and it can be dated in the first half of the 2nd century AD.

## 3. Materials and Methods

The 783 cattle remains under study here filled a pit located in front of the Roman villa of Vilauba. To understand the origin and nature of this exceptional assemblage, a methodology based on a contextual taphonomy approach was used [23], with the integration of archaeozoological variables, stratigraphy and context, and a GIS analysis.

### 3.1. Recording Methodology and Spatial Data Management

The particular nature of the first layer of the pit’s backfill (Stratigraphic Unit 1700) made it advisable to prepare a specific recording methodology in order to answer the many questions posed by the find. The complex depositional sequence of the remains, with multiple superimposed individuals, required a particularly exhaustive and accurate archaeological field record in order to allow the spatial restitution of the deposit and its formation process.

**Figure 5:**
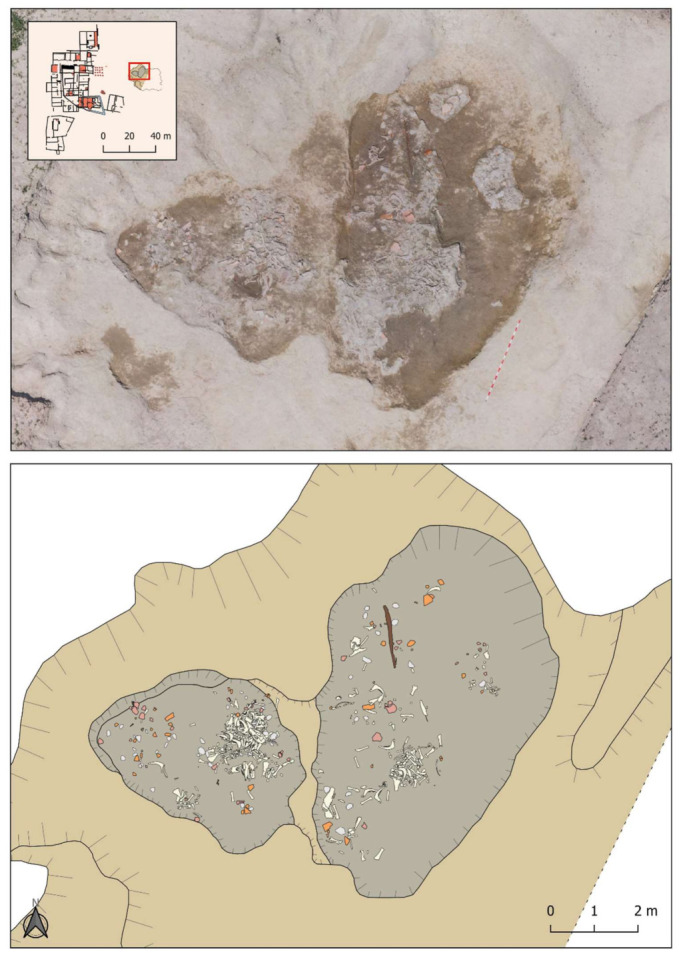
Pit ground plan.

The distribution of the bones dumped in the pit showed that they mainly filled the deepest sectors, with two large cavities inside of which seven smaller groups could be differentiated, individualised correlatively as A-1 to A-7 (Figure 5). Therefore, when the excavation was planned, the dump was subdivided into seven areas. In the case of superimpositions, the areas were sequenced in deposition layers of about 5 cm., which were documented in order from the most recent (Layer 1) to the earliest (Layer 5). Each bone or set of bones, if they had an obvious connection, was given its own record number to allow its subsequent identification in the laboratory. Although the sediment was not sieved, all faunal remains from the pit were recovered, as bone recovering and recording was performed by 5 cm layers that were carefully excavated.

All those bones found at the contact limit between EU 1697 and EU 1700, but that in all probability were already part of the faunal remain dump, were included in the so-called Layer 0. Given their superficiality, those remains that did not in the first instance appear to belong to a concentration were not coordinated.

Apart from the traditional photographs, the graphic documentation of the remains was carried out using orthophotogrammetry, both general and detailed, which was later georeferenced with GIS software (Quantum GIS 3.10), based on the ETRS89 31N datum (EPSG: 25831). The identified bones were then reliably represented by reproducing their order in the assemblage using vector polygons. These were in turn associated with a database or table of attributes in which all archaeozoological information was included (see next section). This facilitated the analysis and spatial management of the data through filtering and intelligent searches, and ultimately made it possible to obtain an overview of the assemblage layout that would have been difficult to achieve without the use of GIS.

### 3.2. Archaeozoological Analysis

The Catalan Institute of Classical Archaeology osteological reference collection was used for identification. The taxonomic variability was based on the relative frequency (NISP) and Minimum Number of Individuals (MNI), following Lyman [24]. The anatomic variability was based on the relative frequency of each element and the Minimum Number of Elements (MNE), following Lyman [25]. Age at death was recorded on the basis of the fusion stage of post-cranial bones, following Barone [26] and the eruption and wear of mandibular teeth. Tooth wear stages follow Grant [27] and were grouped into the age stages suggested by O’Connor [28]. The sexual composition of the population was ascertained by metric analysis. Withers height was estimated using Matolcsi’s criteria [29].

Butchery marks were described as ‘chop’, ‘cut’, and ‘saw’ marks, as they may have been linked to different stages in the carcass processing [30,31,32], when recording their location and orientation. These marks were associated with butchery where possible following [33] and [34].

Fracture types were identified following the criteria in [35]. Individual fractures were categorised as helical (fracture of bone in a fresh state), dry (fractured after loss of moisture and organic content), and new (breaks that occurred during or after excavation).

Biological or natural biostratinomic modifications were identified following [35,36]. We differentiated between weathering, root etching, gnawing, trampling, and abrasion when recording their location and orientation.

## 4. Results

A total of 1058 faunal remains were recovered in Stratigraphic Unit 1700 (Table 1), 783 of which were from cattle. Those are the remains presented here due to their singularity in relation to the other scarce faunal remains recovered in the same stratigraphic unit, in the whole deposit, and in other assemblages excavated in the villa, as we will show below.

As explained in the methodology section, the excavation of this stratigraphic unit was divided into levels and areas in order to document the deposition process and the origin of the remains. Most of the remains were concentrated in Layers 0 and 1 and Areas 0 and 1, filling the irregular V-shaped form of the pit. In taxonomic terms, we observed that the presence of non-cattle species was mainly concentrated in Layer 0 (the layer in close contact with Stratigraphic Unit 1697), being nearly absent in the other layers (Table 1).

### 4.1. Natural Biostratinomic Modifications

Of the remains, 189 (24%) showed some kind of natural alteration on their surface. These alterations were caused by the action of the humic acid contained in plant roots (30 remains), gnawing by carnivorous canines (113 remains), the action of atmospheric agents (43 remains), and the gnawing of rodent incisors (three remains). Therefore, carnivore action was the natural element that most affected the assemblage (14% of the total), with the others being mere testimonial effects.

**Figure 6:**
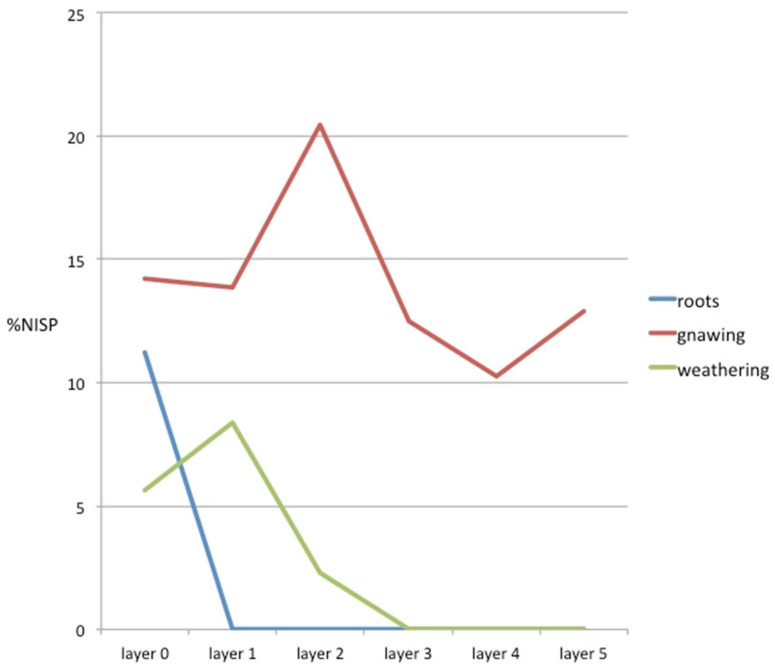
Number of natural modifications documented in the different layers of the Vilauba pit.

Figure 6 shows the distribution of alterations by layers. It can be seen that the remains altered by the action of plant roots were concentrated in Layer 0; i.e., those closest to the surface, and were non-existent in the other layers. The action of carnivores was mainly documented in the first three layers (those nearest the surface and where there was a greater accumulation of remains), although gnawed remains were documented in all the layers (Figure 6). The action of atmospheric agents, in particular water, was concentrated in the first two layers, although with minimal effect. This only affected 6% of the remains in Layer 0, 8% of those in Layer 1, and 2% of those in Layer 2. Rodent action was also documented on three remains in Layer 1. No exfoliations or alterations caused by trampling were documented.

**Figure 7:**
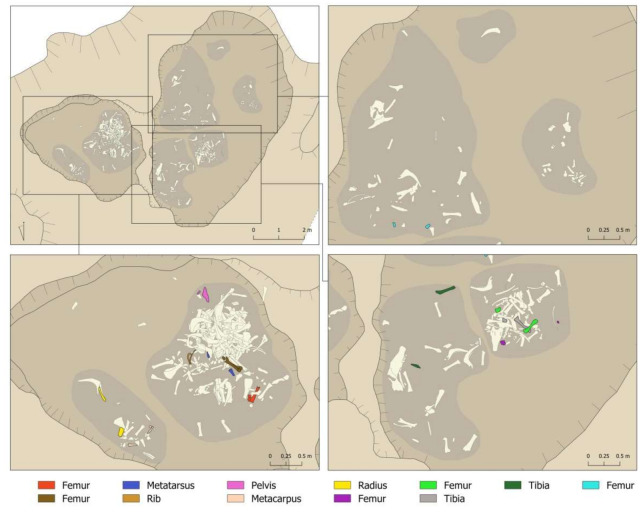
Reassemblages documented in Layer 1 of the different areas of the Vilauba pit.

It was also possible to document the action of the sedimentation on the bones. Five complete cattle heads were recovered from the pit, although it was not possible to clean and study them due to the high risk of fragmentation if the sediment were removed. At the same time, 404 bones (51.6%) presented dry fractures that occurred after the incorporation of the remains in the archaeological deposit. Several refitted fragments were documented in the laboratory, and also showed this breakage through sedimentation. We documented reassemblages in five femurs, one metatarsus, one rib, one pelvis, one metacarpus, one radius, and two tibiae, all in Layer 1 of the different areas (Figure 7). In Figure 7, the horizontal dispersion of these refitted fragments can be seen very clearly. This is fragmentation and dispersion that would have occurred once the pit had been filled. It was not possible to record vertical refitted fragments (between layers) for logistical reasons, as it would have been impractical to spread out so much material at once. However, we understand that just as there were dry fragmentations that led to a horizontal displacement of the remains, there were probably also fragmentations that led to their vertical displacement.

### 4.2. Body Part Representation, Minimum Number of Elements (MNE), and Minimum Number of Individuals (MNI)

In Table 1 we can see the total Number of Remains Identified (NISP) and the Minimum Number of Elements (MNE) of each skeletal element, taking into account the laterality (where it was possible to identify it). The NME makes it possible to determine the taphonomic effects or anthropogenic selection made of an assemblage, as it assumes that all the skeletal elements of an animal were present at the time of its death. Any deviation from this expected skeletal representation would have been caused by post-mortem factors [24].

The first thing we can see is that all the elements of the skeleton are documented. The jaws and the skull (horn cores not included), all the elements that make up the trunk, and all the limbs were documented (Table 2). We can also observe that there are few differences between NISP and MNE, indicating that the elements do not present intensive fracturing/fragmentation. In turn, these elements are present in a fairly homogeneous way, both in terms of NR and especially of MNE, documenting a fairly homogeneous MNI. The humeri indicated the presence of at least 15 individuals; the radii, scapulae, and talus of 14; the femurs and calcaneus of 13; and the pelvis, tibias, metacarpi, and metatarsi of 12.

This representation is also similar among elements with laterality. There were 14 right scapula and 12 left scapula remains; 17 right radius and 16 left radius remains; and 11 right and 12 left metacarpal remains (Table 2).

There are some elements, however, that are less represented or are almost non-existent in the assemblage. These are phalanges, carpal and tarsal bones, patellae, ribs, and vertebrae. For example, only three left kneecaps, four atlases, two axes, and four third phalanges were documented.

**Figure 8:**
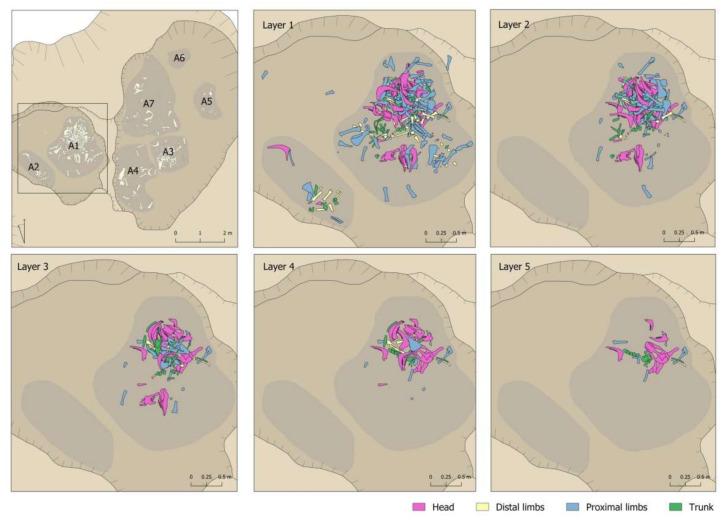
Body-part representation documented in the different layers of Areas 1 and 2 of the Vilauba pit.

**Figure 9:**
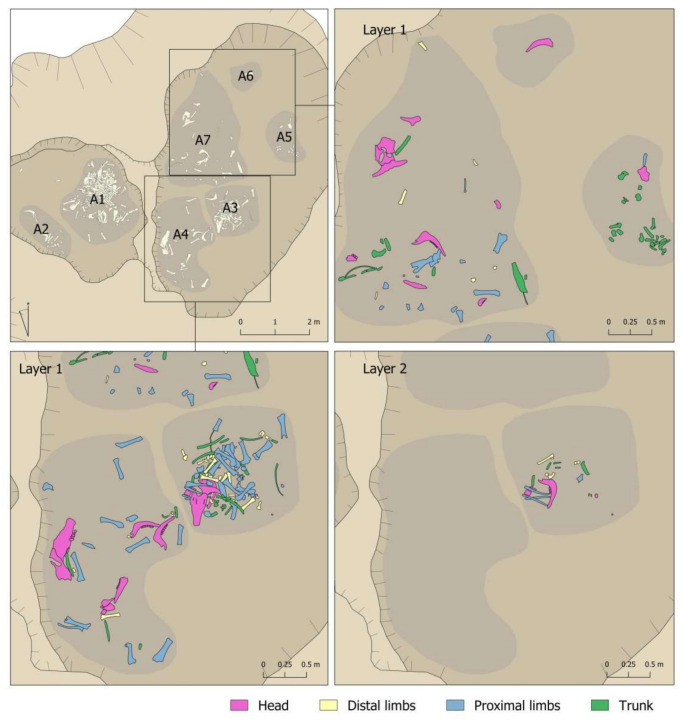
Body-part representation documented in the different layers of Areas 3, 4, 5, 6, and 7 of the Vilauba pit.

It should be emphasised that there was no grouping in the pit by elements or by anatomical parts in each of the sectors (Figure 8 and Figure 9). There was no sector in which remains of the head predominated or where there were more limbs. In fact, in the sector with more remains, there was more of everything. However, the percentage predominance of vertebrae observed in Sector 5 should be noted (Figure 9). This predominance was caused by the documentation of 11 vertebrae that would have been connected to each other, an aspect that will be discussed in more detail in Section 4.6

Nor was documented a remains-deposition sequence according to layers (Figure 8 and Figure 9). For example, there was no indication of an initial deposition of skulls and jaws followed by a deposition of distal parts of limbs, etc. that could be linked to some order in the deposition of the body parts. In this respect, a diversity of anatomical elements and parts was documented in all layers. In this respect, however, we wish to highlight the greater presence of the bones less represented in the assemblage (ribs, vertebrae, phalanges, carpal/tarsal bones) in the lower levels. Level 5 had the largest number of documented vertebrae in proportion to the number of remains recovered. In percentage terms, 29.03% of the remains were vertebrae compared to 12.3% and 6.82% documented in Levels 1 and 2 respectively. A similar relationship can be observed for ribs, where the highest proportion was documented in Level 4 (23.09%), compared to Levels 1 and 2, where they accounted for 14.84% and 19.32%, respectively. The phalanges were concentrated only in the first three levels, but once again with more in the lower level (14.58%) than the two upper levels (5.81% and 4.55% respectively).

### 4.3. Anthropic Modifications

Of a total of 783 cattle bones, 309 bones (39%) were complete, 51 (6.5%) presented new fractures that occurred during excavation or storage and only 22 (2.8%) bore helical fractures (Table 3).

Despite this lack of helical or fresh fractures, 96 cattle bones (12.3%) presented butchery marks (Table 3). These butchery marks were documented on all parts of the skeleton (head, proximal limbs, trunk, and distal limbs) (Table 3). We documented a predominance of cut marks on all the elements; chop marks were infrequent and saw marks non-existent. The skeleton elements with the highest percentage of cut marks were the humeri, phalanges, and scapula. Chop marks were mainly documented on the ribs.

On the basis of their location and orientation, it was possible to establish some of the activities that produced those butchery marks.

In terms of the head, some long, parallel cut marks around the mandibular condyle were documented on one mandible. Such marks could indicate the disarticulation of the jawbone from the head in at least one individual. Cut marks on the dorsal side of a mandible at the level of the third molar were also observed, suggesting the possible detaching of the tongue [33].

**Figure 10:**
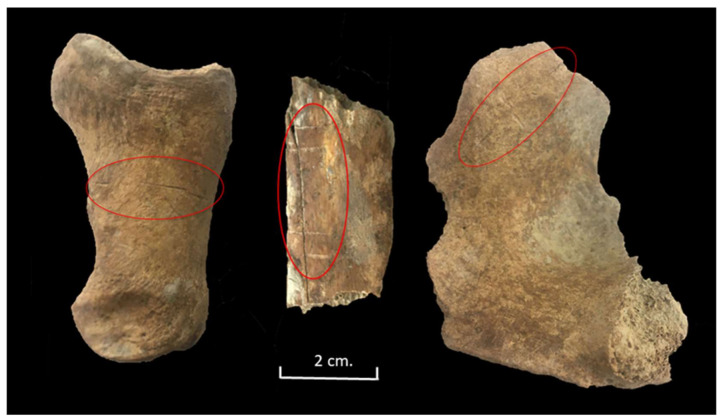
Cut marks documented on a bovine phalange, rib, and atlas from Stratigraphic Unit 1700 of the Vilauba pit.

At least one example indicated that the individual had been decapitated. A long cut mark (blade insertion) behind the ventral aspect of the cranial articular surface of an axis suggests that the head was extended to expose the neck and a blade inserted to decapitate the head when the animal was on the ground (Figure 10).

Very fine knife cuts were observed on the proximal epiphysis of a metacarpal and on seven metatarsals. These marks have been linked to the skinning of the carcass, although they could also have occurred when the ligaments were cut to detach the feet [33,34]. Eleven phalanges showed cut marks, which may be associated with skinning (Figure 10).

**Figure 11:**
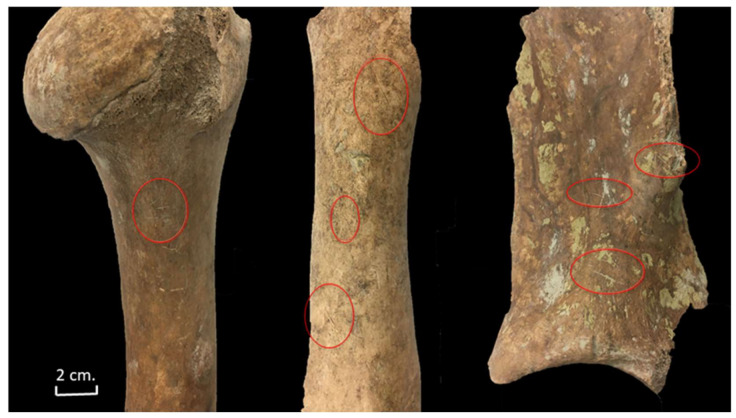
Cut marks documented on a cattle humerus, femur, and scapula from Stratigraphic Unit 1700 of the Vilauba pit.

All the long bones, especially the humerus, showed cut marks on the diaphysis (essentially on the medial shaft of the diaphysis) (Figure 11). These knife cuts could have occurred during meat removal and/or filleting. Although some chop marks were also documented on the diaphysis of seven long bones, they would never have led to the fracture of those bones.

Cut marks, as well as some chop marks, were documented on eight scapulae, showing that small and large blades were used to disarticulate the scapula and remove the meat. These marks were mainly documented on the medial side of the distal end of the scapula and also on the distal side of the spine (Figure 11).

Different butchery marks were also documented on 11 ribs (Figure 10). We observed cut marks on the lateral side of four corpuses that could have been the result of filleting. At the same time, some cut marks observed on the medial side of two corpuses could indicate the evisceration of those carcasses. Three ribs were chopped, and some chop marks were documented on the corpus of five ribs, which could indicate that the meat attached to the ribs was divided into portions.

This butchery process, however, did not involve the breaking or sawing of bones. No thermal alteration of the bones was observed.

### 4.4. Ages at Death

As can be seen in Table 2, the best-represented element was the jaw, with at least 17 right and 13 left jaws. The ages estimated from dental wear indicate that these 17 right jaws corresponded to individuals with the following ages when they were slaughtered: one individual was between 2 and 3 years old; two were between 3 and 6; 12 were between 6 and 8; and two were between 8 and 10. Estimating age through dental wear on the 13 left jaws showed very similar results: two individuals between 3 and 6 years of age and 11 individuals between 6 and 8 (Table 4).

As far as the estimate of age from the state of long bone fusion is concerned, fused remains, mainly of elements that fused after 20–24 months and later, predominated. At the same time, unfused remains from elements that fused before 24–30 months were almost non-existent (Table 5).

These data indicate a consistent prevalence of adult specimens, both from long bones and mandibles.

### 4.5. Wither Height and Sex

It was possible to calculate various heights at the withers from the maximum length of the long bones that were whole and fully fused (Table 6). As can be seen in Table 6, there was a certain recurrence of withers heights. Based on the withers heights of nine right metacarpi, all available withers heights from different elements were grouped together. Using indices to derive withers height is not exact, as there can be variations from different elements, but this data could suggest the presence of some 14 different individuals.

**Figure 12:**
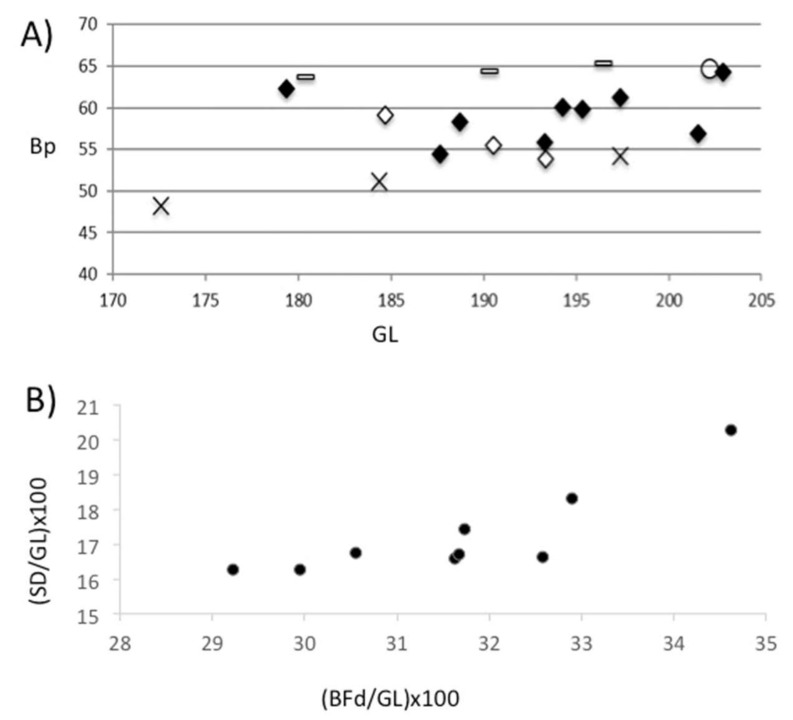
(**A**) Scatter diagram of GL (greatest length) and Bp (greatest width of the proximal epiphysis) measurements (in cm) of cattle from the Vilauba pit (black rhombus) and from other early Roman contexts in the villa (white rhombus), compared to three males (line), three females (cross), and one castrate (circle) from the Camargue cattle breed. (**B**) Scatter diagram of (BFd (distal width across both condiles)/GL (greatest length)) × 100 and (SD (minimum diaphysial width)/GL (greatest length)) × 100 indices of cattle from the Vilauba pit.

In order to document whether this variability in withers heights could have been due to the presence of females, males, and castrates, an attempt was made to assess the gender of the individuals represented in the assemblage. An osteometric approach was used, bearing in mind that males tend to have more robust bones than females, and that castrates tend to have bones as robust as males but longer [37,38]. The maximum length and width of the proximal diaphysis of the nine right metacarpi that could be calculated at the height of the withers were compared with the same measurements of three males, three females and one castrate of the rustic breed from the Camargue (south of France), [38] and with another three Vilauba individuals from other early Roman contexts (Figure 12A). At the same time, the established method for sexing cattle metacarpals presented in Davis et al. [39] was also applied (Figure 12B).

Figure 12A shows that most of the individuals from the pit were situated between the males and the two tallest females. There was also one individual that could clearly be associated with a modern castrated individual. Figure 12B shows similar results. There were three individuals that could be considered females, five individuals that could be considered males, and one castrated individual (Figure 12B). Therefore, these nine right metacarpi could have corresponded to male and female individuals, and even some castrated individuals. Figure 12A also shows that the individuals in the pit would have been of a similar size to those recovered in other early Roman contexts of the villa.

### 4.6. Articulating Elements

Several articulated bones were recovered during excavation, although no whole articulated skeletons were documented. At the same time, other connections were recorded in the laboratory. In total, it was possible to document connections between 82 elements (10.3%). These articulations were predominantly between distal limb elements, although some were between vertebrae and long bones (Table 7). It was also possible to match 11 right and left mandibles, taking into account the wear stage of their teeth and osteometry (Table 7).

**Figure 13:**
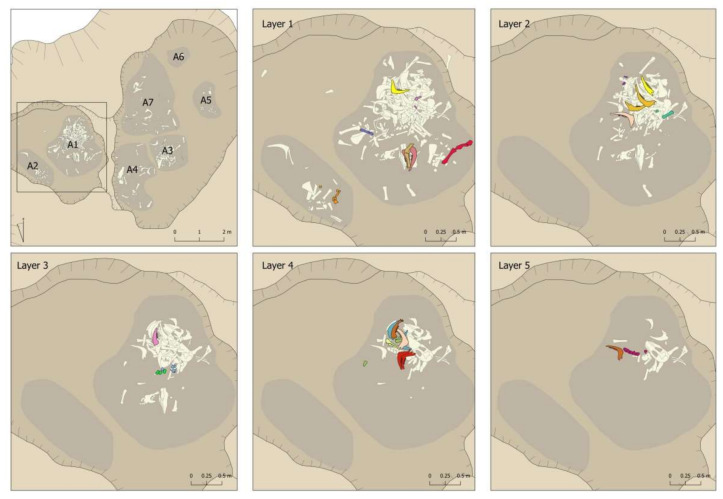
Connections documented in the different layers of Areas 1 and 2 of the Vilauba pit.

**Figure 14:**
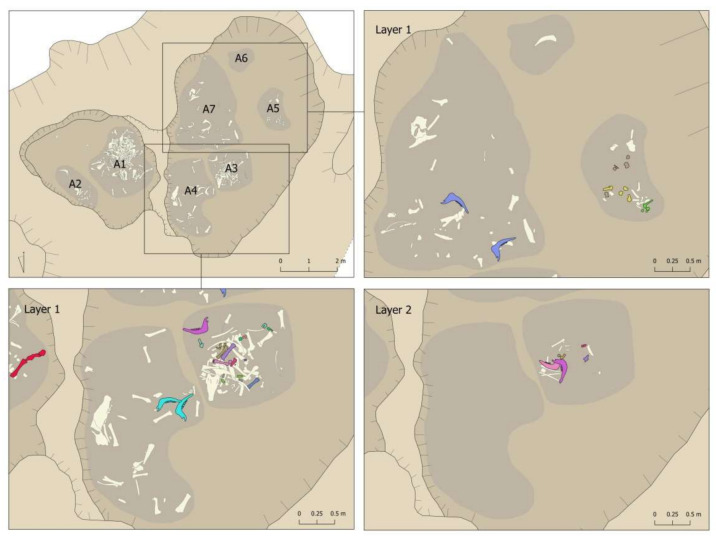
Connections documented in the different layers of Areas 3, 4, 5, 6, and 7 of the Vilauba pit.

Figure 13 and Figure 14 show a detailed representation of these articulations by areas and layers in the pit. The first issue to point out is that connections were documented in all areas and layers, with a predominance in those areas and layers with the most remains (Area 1 and first layers of the different areas). The second issue to highlight is that all the connections were documented within the same area. The only exception was the connection between a right mandible documented in Area 1 (Layer 3) and a left mandible documented in Area 3 (Layer 2) (pink mandibles in Figure 13 and Figure 14). In contrast, we documented connections between layers, such as the yellow mandibles from Area 1 or the lilac radius + ulna connection from Area 3 (Figure 13 and Figure 14). This suggests a certain degree of horizontal as well as vertical movement of the remains after their deposition in the pit.

### 4.7. Potential Edible Meat

In order to estimate the amount of meat that could have been obtained from the carcasses of these 14 individuals, the potential edible meat was calculated (Table 8) by comparing the withers heights of the animals with unimproved animals of a similar size [40,41]. These provided living weight estimates from which meat yields were calculated using two different evaluations [40,42].

These estimates show that between 1.3 and 2 tons of meat could have been obtained from approximately 14 individuals, which could have fed some 2600 people at a time (500 g per person).

## 5. Discussion

The detailed archaeozoological and GIS analysis shown above, combined with the information obtained from the stratigraphic record, allowed us to consider some hypotheses regarding the origin and formation of this assemblage of cattle remains.

The excavated archaeological stratigraphy showed that the bone remains were dumped immediately after the clay had been extracted, as the layer of bones (U.S. 1700) was deposited directly on the bottom of the pit (Figure 4, Phase 2A). With regard to the formation of the deposit itself, we propose that this was carried out unitarily over a very short period of time, as evidenced by the absence of colluvial levels between the bone layers. The study of the natural and anthropic modifications presented here corroborated this statement, as all the remains presented a similar taphonomic history and were affected by the same biological agents (gnawing). The general absence of weathering (which only affected 5% of the assemblage) and total absence of sedimentary abrasion and trampling in all layers and areas also showed that this assemblage did not have a complex taphonomic history, but quite the opposite.

The fact that some of the remains of the most superficial layers of the deposit were altered by the action of water and roots could be an indication that these last layers were in the open air for a longer period before being permanently covered. The presence of bone remains from other species in the surface level (Layer 0), resulting from the habitual consumption of the villa’s inhabitants, would also fit this interpretation. It is also supported by the stratigraphy itself, since the bone remains were covered by colluvial clay (Figure 4, Phase 2B). Shortly afterwards, when the bones had been mostly covered by clay and vegetation, the pit was permanently filled with construction and consumption remains and other debris (UEs 1697, 1696). The most modern finds in these stratigraphies allowed us to place their formation around the last decade of the 1st century AD.

Therefore, based on this evidence, we suggest that the cattle assemblage under study here was a homogeneous deposit. The presence of several articulated partial skeletons documented during and after excavation in all layers and areas of the deposit suggests that the context in which they were found is a primary deposit. This fact is also shown by the lack of diverse natural alterations in the remains, indicating that they did not come from different assemblages with different taphonomic histories, but quite the opposite.

We therefore propose that we are looking at a homogeneous assemblage in a primary position that was dumped in the pit during a brief time. During that time, however, canines gained access to the remains and gnawed on a few of the elements (113 remains). However, we considered that the impact of carnivores on the assemblage is not only evident from the marks they left on the remains, but also from the fact that connections were documented during the laboratory work that had not been observed in the field and were a result of the displacement of the remains by the canines when they accessed the pit. On the other hand, the documented anatomical representation is quite homogeneous among the long bones, jaws, pelvises, scapulae, and skulls. In contrast, there are certain elements that are underrepresented, mainly at those upper levels. They are those with a lower structural density, such as the vertebrae and ribs [43,44,45,46], or smaller bones such as carpal or tarsal bones, phalanges, patellae, etc.

Given these considerations, we consider that the anatomical representation documented in the pit was not just the result of human intervention (such as the result of skinning). We also consider that dogs gnawed some bones, displaced others, and also could have been responsible for a few of them disappearing altogether once ingested. These would have been mainly the smaller, lower-density bones from the upper levels. The action of dogs on animal-remain deposits, resulting in a differential preservation due to the ingestion of less dense and/or smaller bones, is well documented archaeologically [47,48,49].

If we assume that gnawing, together with human action, could have contributed to the biases in the assemblage, we can propose that this pit was filled with whole cattle skeletons. The fact of having documented connections throughout the deposit, a similar number of right and left elements, together with a similarity of ages between the right and left jaws and the long bones, allowed us to propose that all these elements would have corresponded to the skeletons of some 14 individuals.

They were predominantly males and females, although we also documented the presence of castrates, with a withers height of between 111 and 140 cm (average 123 cm). These individuals had slightly higher withers heights than those documented in other contexts of the villa, ranging from 107 to 122 cm. The study of age at death shows that they were all adults when they died, with a predominance of 6- to 8-year-old individuals, except for one individual that was 2–3 years old and two individuals that were 8–10 years old. These ages at death are similar to the slaughter patterns documented during the early Roman phase of the villa, with a clear predominance of animals slaughtered at adult and older ages [20,50]. Therefore, these ranges of ages would be the predominant cattle ranges of age present at the villa. Thus, considering the profiles of the 14 individuals excavated, we could conclude that most of these animals were probably part of the villa’s workforce (draught animals, but also for breeding and traction). It must be highlighted that two metatarsals and one metacarpal were present an enlargement of the medial trochlea, which can occur in those animals repeatedly exploited as draught animals [51].

We were also able to document that these carcasses were dumped in the pit after being processed, as shown by the fact that a fairly high number of marks were recorded (on 12.3% of the remains). The study of the type of mark and their location and orientation revealed that, once slaughtered, the individuals had probably been beheaded, stripped, eviscerated, partially dismembered, and skinned. All this, however, was carried out without fracturing the bones.

There are many studies that demonstrate how the processing of carcasses in Roman times followed fairly standard guidelines [33,52,53,54,55,56]. It has been suggested that, once the head had been removed, the fore and hind limbs were the first parts to be detached from the body, leaving the rib cage, vertebrae, and pelvis as a unit. After removing the limbs, the carcass was split. Final processing would have taken place to reduce the carcass to portions suitable for cooking [34]. The most widely used tool was the cleaver, a large butcher’s knife used for intensive slicing and dismemberment to maximise the amount of meat obtained from each individual, cutting the bones to quarter and dismember the carcass [52,53,54,55,56].

Therefore, we propose that some 14 cattle carcasses were processed in a short time, but not in the usual way documented in other assemblages excavated at the villa interpreted as waste from culinary preparation and consumption [20], nor as the usual way proposed for Roman times [33,52,53,54,55,56]. We can rule out the possibility that we are dealing with a classic waste dump from domestic meat consumption. Neither does it appear to be butchery waste since, the possible bias caused by the dog actions notwithstanding, the assemblage under study is made up of elements of the whole skeleton. Heads, the distal and proximal parts of limbs, and trunk elements were documented. Neither is it a deposit formed by the waste from activities linked to hide preparation, bone and antler working, or glue manufacture. As explained in the introduction, several studies propose that such deposits consist of specific elements with certain fracturing patterns that were not documented here. Moreover, that type of waste is usually generated in urban contexts where there is a large agglomeration of people.

The characteristics and nature of the Roman villa of Vilauba, a rural establishment with modest residential buildings and various facilities intended for the agricultural exploitation of the most immediate territory, with a maximum estimated population of between 25 and 30 people, leads us to rule out the option that these cattle were slaughtered for the inhabitants’ own consumption. Likewise, the hypothesis of a banquet or large celebration for hundreds of invitees seems equally unlikely if we take into account the mainly agricultural nature of the establishment and the settlement structure in this territory, which was marked by small scattered rural establishments and a single urban nucleus, Gerunda, certainly of very modest size. The biomass calculation carried out in Section 4.7 shows that between 1.3 and 2 tons of meat could have been obtained from these 14 individuals. This could have fed from some 2600 people (500 g per person) until several thousand people (100 g per person) at a time, a totally ruled out possibility if we take into account the territory in which the villa was located. The constructive and architectural characteristics of the urban part of the Vilauba villa, very far from the models of other Roman villas with a clearly residential vocation, with numerous rooms decorated with mosaic pavements and rich pictorial decorations, or large reception and representation rooms, also do not suggest the possibility of large-scale celebrations. In this same sense, the absence of any ritual evidence or ritual connotation in the assemblage (as has been explained in Section 2.1), as well as of any other indicator of a cultic nature in the pit itself or in the settlement, leads us to rule out a possible interpretation of the carcasses as a commemorative or religious deposit.

Another option is that the slaughter of these 14 individuals was not due to an action planned over time, as it involved a large quantity of meat being suddenly available. One possibility is that they may have contracted some kind of disease, and it was decided to slaughter them in order to take advantage of their meat. The Latin agronomists describe many diseases as having been common in cattle, including indigestion, stomach aches, dysentery, infections, fevers, coughs, abscesses, lameness, scabies, ulcers, and wounds (Columella, De re rust., I, VI, 1–19; Paladius, Op. agr., XIV). The majority of these illnesses do not leave traces in the bones. In this case, we propose that they would have contracted a disease that would have allowed most of their meat to be taken advantage of. This possible disease would have affected those cattle that predominated in the villa: mainly males and females aged between 3 and 10 years. If we accept this possibility, the only way to use so much meat (between 1.3 and 2 tons) would have been to preserve most of it. A number of techniques (e.g., salt curing, smoking, marinating, drying) were used to preserve foodstuffs. In the case at hand here, the meat preservation would clearly have been carried out without the bone and after a meticulous defleshing. This would have allowed the recovery of large amounts of meat, and would also explain the presence of fine marks on most of the bones and the absence of fractures.

Columella describes a dry process for preserving meat in which a pig is boned that could have been the technique used at Vilauba to preserve such a huge quantity of meat. Salt was rubbed all over the pieces and into the cavities from where the bones had been removed. Then the pieces were pressed between weighted boards for three days to extract as much moisture as possible. The pieces continued to be rubbed with salt and a little saltpetre for 9–12 days, then rinsed and hung to dry (Rust. 12.55.1–4). This is the artisanal method used to make ham today. With all this preserved meat, the inhabitants of Vilauba would have had several years’ supply to add to their everyday meals, which mainly consisted of cereals and vegetables, as well as a surplus to sell to the markets.

## 6. Conclusions

In this article, we have presented an exceptional assemblage of cattle remains. The detailed archaeozoological study combined with the GIS analysis and the information obtained from the stratigraphic record was key to a correct interpretation of this assemblage. This approach allowed us to propose that this was a homogeneous deposit in primary position that had been dumped in the pit over a short time. The study also allowed us to observe that this deposit consisted of the carcasses of some 14 adult bovines that had been processed in a very different way to the usual procedure and as a result of a situation forced by a specific circumstance.

Based on all the data presented, we consider the possibility that these individuals may have been slaughtered due to an accident or illness, and that most of their meat may have been preserved as the only means of taking advantage of such a large quantity. Therefore, we present a baseline for identifying this kind of preserve in other sites and assemblages.

Whatever the specific event that led to the formation of this assemblage, all the information revealed in this study inclines us to propose that it was a specific event brought about by exceptional circumstances that led to an unusual use of these carcasses. Furthermore, the pit in front of the villa from which clay had been extracted was considered as the most suitable place to dispose of such a large number of cattle carcasses.

It was the excavation of an area outside the villa, a very rare circumstance, that allowed us to open this window onto a very specific and exceptional moment in the life of Vilauba’s inhabitants.

We would like to end by emphasising the importance—hinted at by this study—of cattle in Vilauba, whether as draught animals and/or for breeding, both of which were documented at the villa. The find of this pit presupposes the existence of at least 14 individuals (males, females, and castrated animals) living at the same time in Vilauba or in its immediate surroundings.

Further research is needed. It would be of prime interest to investigate the many wider socio-economic implications regarding the presence of these animals at the site. At the same time, a pathogen ancient DNA analysis of these 14 individuals would be a suitably conclusive test for the hypothesis presented here.

## Figures and Tables

**Table 1:** Distribution of faunal remains from Stratigraphic Unit 1700 of the Vilauba pit by areas and layers.

Areas	Layers	Cattle (*Bos taurus*)		Sheep/Goat (*Ovis/Capra*)		Pig (*Sus domesticus*)		Equid (*Equus sp.*)		Dog (*Canis familiaris*)		Red Deer (*Cervus elaphus*)		Birds		Malacology		Total
		NISP	%	NISP	%	NISP	%	NISP	%	NISP	%	NISP	%	NISP	%	NISP	%	
Area 0	Layer 0	267	34.1	91	92.9	39	90.7	19	63.3	2	100	2	66.7	1	33.3	2	100	423
Area 1	Layer 1	105	13.4	1	1.02	0	0	1	3.33	0	0	1	33.3	0	0	0	0	108
	Layer 2	56	7.15	4	4.08	0	0	0	0	0	0	0	0	2	66.7	0	0	62
	Layer 3	48	6.13	0	0	0	0	4	13.3	0	0	0	0	0	0	0	0	52
	Layer 4	39	4.98	0	0	0	0	0	0	0	0	0	0	0	0	0	0	39
	Layer 5	31	3.96	0	0	2	4.65	4	13.3	0	0	0	0	0	0	0	0	37
Area 2	Layer 1	27	3.45	1	1.02	1	2.33	0	0	0	0	0	0	0	0	0	0	29
Area 3	Layer 1	75	9.58	0	0	0	0	0	0	0	0	0	0	0	0	0	0	75
	Layer 2	32	4.09	0	0	0	0	0	0	0	0	0	0	0	0	0	0	32
Area 4	Layer 1	28	3.58	0	0	0	0	2	6.67	0	0	0	0	0	0	0	0	30
Area 5	Layer 1	25	3.19	0	0	0	0	0	0	0	0	0	0	0	0	0	0	25
Area 6	Layer 1	1	0.13	0	0	0	0	0	0	0	0	0	0	0	0	0	0	1
Area 7	Layer 1	49	6.26	1	1.02	1	2.33	0	0	0	0	0	0	0	0	0	0	51
Total	Total	783	100	98	100	43	100	30	100	2	100	3	100	3	100	2	100	964

**Table 2:** Number of Identified Remains (NISP), Minimum Number of Elements, and Minimum Number of Individuals for cattle elements recovered in the Vilauba pit.

ELEMENTS	NR				MNE		MNI
	RIGHT	LEFT	NO DET.	TOTAL	RIGHT	LEFT	
SKULL	11	11	12	28	10	10	10
MANDIBLE	26	15	3	44	17	13	17
SCAPULA	14	12	3	29	14	12	14
HUMERUS	21	14	0	38	15	13	15
RADIUS	17	16	1	36	14	12	14
ULNA	11	8	0	19	10	8	10
PELVIS	17	13	11	41	11	12	12
FEMUR	24	23	10	57	13	11	13
TIBIA	15	22	0	37	9	12	12
PATELLA	1	3	1	5	1	3	3
METACARPUS	12	9	6	27	12	9	12
III CARPAL	3	1	0	4	3	1	3
IV CARPAL	2	2	1	4	2	2	2
INTERMEDIAT CARPAL	1	1	0	2	1	1	1
RADIAL CARPAL	2	4	0	6	2	4	4
ULNAR CARPAL	2	3	0	5	2	3	3
METATARSUS	11	12	7	30	11	12	12
ASTRAGALUS	15	13	0	28	14	13	14
CALCANEUS	13	12	0	25	13	12	13
CENTROQUARTAL	4	8	0	12	4	8	8
II TARSAL	1	0	0	1	1	0	1
III TARSAL	0	1	0	1	0	1	1
PHALANX 1	18	11	2	31	18	11	2
PHALANX 2	8	8	0	16	8	8	1
PHALANX 3	2	2	0	4	2	2	1
RIBS	1	4	118	123			
SACRUM				8			8
ATLAS				4			4
AXIS				2			2
CERVICAL				21			3
THORACIC				33			3
LUMBAR				18			3
CAUDAL				2			1

**Table 3:** Number of cattle remains with different recorded butchery marks, helical fractures, and dry fractures documented in the Vilauba pit.

ELEMENTS	Cut	Chop	Saw	Helical Fractures	Dry Fractures
SKULL	0	0	0	0	22
MANDIBLE	5	1	0	3	24
SCAPULA	8	3	0	0	19
HUMERUS	13	2	0	3	15
RADIUS	7	1	0	1	12
ULNA	1	0	0	0	11
METACARPUS	1	1	0	3	9
PELVIS	1	1	0	1	39
FEMUR	7	3	0	0	26
TIBIA	5	1	0	2	22
METATARSUS	7	0	0	3	9
ASTRAGALUS	2	0	0	0	3
CALCANEUS	2	1	0	0	5
CARPALS/TARSALS	0	0	0	0	7
PHALANGES	11	0	0	0	3
RIBS	6	5	0	3	123
VERTEBRA	0	1	0	3	55
TOTAL	76	20	0	22	404

**Table 4:** M3 wear stage and estimated age of right and left mandibles recovered from the Vilauba pit.

Age	M3 Wear Sage	No. of Right Mandibles	No. of Left Mandibles
2–3 years	B	1	0
3–6 years	G	2	2
6–8 years	J	2	1
	K	8	7
	L	2	3
8–10 years	M	2	0

**Table 5:** Number of cattle with unfused, in the process of fusion, and fused remains recovered from the Vilauba pit (p = proximal; d = distal).

ELEMENTS	Unfused	In Process	Fused	Age at Fusion
Radius, p	0	2	19	12–15 months
Humerus, d	1	0	19	15–20 months
Phalange I	0	0	31	20–24 months
Tibia, d	0	0	6	24–30 months
Metapodial, d	1	0	36	24–30 months
Femur, p	6	0	6	36–42 months
Humerus, p	2	2	7	24–30 months
Radius, d	7	0	17	24–30 months
Femur, d	18	1	14	24–30 months
Tibia, p	10	2	7	24–30 months

**Table 6:** Cattle withers heights (in cm) calculated on the basis of the length of several skeletal elements from the Vilauba pit (MC = metacarpus, MT = metatarsus, HU = humerus, FE = femur, RA = radius, TI = tibia, r = right, l = left).

Withers Height	ind 1	ind 2	ind 3	ind 4	ind 5	ind 6	ind 7	ind 8	ind 9	ind 10	ind 11	ind 12	ind 13	ind 14
	111	115	116	117	119	120	121	122	124	125	128	133	135	140
MC, r	111.1		116.2	116.8	119.7	120.3	120.9	123.1	124.8	125.6				
MC, l								123.1						
MT, r				117.6	118.8				124.2		129.1	133.4		
MT, l	111.9					120.1		123.3	124.8	124.9	129	133.9		
HU, r	111													
HU, l								122.3						
FE, r			116.5								127.8			
FE, l														
RA, r		115.7		117.5				122.3	124.4					
RA, l					119.5						128.4			
TI, r														140.6
TI, l		114.4								125		132	134.9	

**Table 7:** Connections between cattle remains documented in the different areas of the Vilauba pit. Figure 13. and 14 for the colour-coding.

Areas	Mandibles	Fore Limb	Trunk	Hind Limb
Area 1	right mand. + left mand.	radius + 2 carpal bones + mc	7 vl	astragalus + calcaneus
	right mand. + left mand.	radius + 5 carpal bones + mc	3 vl + sacrum	femur + pelvis
	right mand. + left mand.	mc + ph1	3 vl	2 tarsal bones
	right mand. + left mand.	humerus + radius + ulna		
	right mand. + left mand.			
	right mand. + left mand.			
	right mand. + left mand.			
	right mand. + left mand.			
Area 2		4 carpal bones + mc		
Area 3	right mand. + left mand.	2 carpal bones + mc + ph1		astragalus + calcaneus
		mc + ph1		tarsal bone + mt + 2 ph1 + ph2
		radius + ulna		tarsal bone + mt + ph1
				astragalus + calcaneus
				ph1 + ph2
				ph1 + ph2
				calcaneus + tarsal bone
Area 4	right mand. + left mand.			
Area 5			4 vc	
			4 vt	
			3 vt	
Area 7	right mand. + left mand.			

**Table 8:** Carcass weight, meat yield, and edible meat for rustic cattle breeds raised in traditional production conditions (information in kg).

MNI	Carcass Weight (Siracusano 2002 [40])	Carcass Weight (Chaix 1976 [41])	% Meat Yield (Siracusano 2002 [40])	% Meat Yield (Ensminger 1973 [42])	Edible Meat (Siracusano 2002 [40])	Edible Meat (Ensminger 1973 [42])
1	225	250	42	60	94.5	150
14	3150	3500	42	60	1323	2100

## Data Availability

Data supporting the reported results can be found at “Repositori de dades de recerca de Catalunya” https://dataverse.csuc.cat/dataverse/ICAC-CERCA, accessed on 1 July 2021.
